# 50 years of rice breeding in Bangladesh: genetic yield trends

**DOI:** 10.1007/s00122-023-04260-x

**Published:** 2023-01-21

**Authors:** Niaz Md. Farhat Rahman, Waqas Ahmed Malik, Md. Shahjahan Kabir, Md. Azizul Baten, Md. Ismail Hossain, Debi Narayan Rudra Paul, Rokib Ahmed, Partha Sarathi Biswas, Md. Chhiddikur Rahman, Md. Sazzadur Rahman, Khandakar Md. Iftekharuddaula, Steffen Hadasch, Paul Schmidt, Md. Rafiqul Islam, Md. Akhlasur Rahman, Gary N. Atlin, Hans-Peter Piepho

**Affiliations:** 1 Bangladesh Rice Research Institute (BRRI), Gazipur, Bangladesh; 2 Institute of Crop Science, Biostatistics Unit, University of Hohenheim, Fruwirthstrasse 23, 70599 Stuttgart, Germany; 3 Shahjalal University of Science and Technology, Sylhet, Bangladesh; 4 International Rice Research Institute (IRRI), Dhaka, Bangladesh; 5 Bill & Melinda Gates Foundation, Seattle, USA

## Abstract

To assess the efficiency of genetic improvement programs, it is essential to assess the genetic trend in long-term data. The present study estimates the genetic trends for grain yield of rice varieties released between 1970 and 2020 by the Bangladesh Rice Research Institute. The yield of the varieties was assessed from 2001–2002 to 2020–2021 in multi-locations trials. In such a series of trials, yield may increase over time due to (i) genetic improvement (genetic trend) and (ii) improved management or favorable climate change (agronomic/non-genetic trend). In both the winter and monsoon seasons, we observed positive genetic and non-genetic trends. The annual genetic trend for grain yield in both winter and monsoon rice varieties was 0.01 t ha^−1^, while the non-genetic trend for both seasons was 0.02 t ha^−1^, corresponding to yearly genetic gains of 0.28% and 0.18% in winter and monsoon seasons, respectively. The overall percentage yield change from 1970 until 2020 for winter rice was 40.96%, of which 13.91% was genetic trend and 27.05% was non-genetic. For the monsoon season, the overall percentage change from 1973 until 2020 was 38.39%, of which genetic and non-genetic increases were 8.36% and 30.03%, respectively. Overall, the contribution of non-genetic trend is larger than genetic trend both for winter and monsoon seasons. These results suggest that limited progress has been made in improving yield in Bangladeshi rice breeding programs over the last 50 years. Breeding programs need to be modernized to deliver sufficient genetic gains in the future to sustain Bangladeshi food security.

**Supplementary Information:**

The online version contains supplementary material available at 10.1007/s00122-023-04260-x.

## Introduction

Rice and food security are synonymous in Bangladesh (Brolley 2015). For more than 166.5 million people in Bangladesh, rice is the main staple food, contributing 97% of total food grain production (BBS 2019; FPMU 2020). Bangladesh ranks fourth among countries globally in rice consumption, with an annual per capita availability of rice of about 213.5 kg (FPMU Database 2020).

Rice is Bangladesh’s largest crop, occupying about 76% of the total cropped area (15.44 million hectares), of which, in 2019–20, about 88% was planted to modern varieties, with traditional landrace varieties covering 12% (BBS 2019). The current intake of rice is about 367 g capita^−1^ day^−1^, which provides approximately 60% of total calories and 50% of total protein for adults (HIES 2016). Nearly 48% of rural workers are directly or indirectly involved in rice production for their livelihood. Rice is grown on more than 13 million farms on approximately 11.77 million hectares, summed over the winter (dry) and monsoon (wet) seasons (DAE 2020). The contribution of rice to the value of the crop sub-sector is about 70% (Mottaleb and Mishra 2016).

Since the Green Revolution of the 1960s, rice output quadrupled in Bangladesh, increasing from 9.67 million tons in 1971 to 38.70 million tons in 2019, with national average yield more than doubling, from 1.50 to 3.29 t ha^−1^ during the same period (BBS 1972; DAE 2020). Key factors underlying these improvements were the government’s support for mechanization and irrigation, controlling fuel and fertilizer prices, improved credit policies, well-organized fertilizer supply, increased quality seed supply by public and private sectors, expansion of the high-yield irrigated winter rice system, and genetic improvements for both favorable and stress-prone environments. With the steady yield growth of recent years, Bangladesh has been self-sufficient in rice production since 2012 (Bell et al. 2015).

In 2050, the population of Bangladesh is predicted to reach 215.4 million. An estimated 44.6 million tons (MT) of milled rice will be required to feed the increasing population of the country (Kabir et al. 2015). At the same time, rice production is hampered by decreasing arable land, increasing climate vulnerabilities like drought, salinity, flood, heat and cold, tidal submergence, water stagnation, and seawater intrusion, all of which pose long-term threats to the country’s agricultural sector and can hinder food security. Climate change is a great challenge for sustaining Bangladeshi rice production and future food security (World Bank 2016).

Over the last 50 years, the Bangladesh Rice Research Institute (BRRI) has developed and released new varieties and conducted variety trials to assess their performance. Within this period, yield has increased due to improved management (agronomic trend) and plant breeding efforts (genetic trend) (Masuka et al. 2017; Rife et al. 2019). Genetic gain can be defined as the rate of increase in performance over a period of time that is achieved through artificial selection and breeding programs (Xu et al. 2017). Over the last 10 to 15 years, there have been several studies assessing long-term genetic gains in different crop species in different countries (Kumar et al. 2021; Laidig et al. 2014, 2017; Rife et al. 2019). To quantify the increase in performance with time that is due to breeding, agronomic and genetic gain need to be dissected, which can be done using models that regress varietal performance for traits of interest on year of release, in trials conducted across locations and years. In “ERA” studies, all varieties in the series are included in the same trials (Duvick et al. 2004; Duvick 2005). Other models allow genetic trend to be estimated from ongoing varietal performance trials that do not test all varieties in all years, but that retain a sufficient number of common checks from year to year to allow genetic and non-genetic trends to be estimated (Mackay et al. 2011; Piepho et al. 2014). It is to be expected that breeding and varietal selection are not the only relevant factors driving crop yields gains when comparing cultivar performance and genetic advances across several locations and years (Ahrends et al. 2018; Muralidharan et al. 2019).

Modern rice research in Bangladesh started in the 1960s, but long-term genetic gain of rice in the country has not yet been quantified. Therefore, the objective of this study was to use historical yield data from trials of BRRI’s released rice varieties to assess the genetic progress achieved due to rice breeding. The key objective of this study was to estimate the long-term rate of genetic improvement delivered by BRRI breeding efforts.

## Materials and methods

### Long-term multi-environment trials (BRRI rice stability trials)

Rice is cultivated in Bangladesh in the monsoon (roughly June-October) and winter (November -April) seasons. The seasons differ greatly in growing conditions and consequently require different varieties, however, both seasons are equally important. Winter rice, grown in the dry season, is irrigated. During the winter, evaporative demand and solar radiation are high, and relative humidity and disease pressure are low. However, rice grown in the monsoon season is rainfed (low solar radiation, high relative humidity, high pest, and disease pressure). BRRI operates large, separate breeding pipelines for each season. BRRI has been conducting multi-location rice variety trials since 2001 for both seasons. One of the major breeding objectives in connection with farmers’ needs is that varieties developed for favorable environments should also have the capabilities of performing well in less favorable environments. Similarly, stress-tolerant varieties should also perform well under non-stress conditions. For example, a flash flood tolerant variety should exhibit high yield whenever there is no flash flooding in a particular area of a farmer’s land. BRRI has 11 regional stations, all having favorable lands where the trials were performed. Conducting trials under on-station conditions has generated good quality data. All trials were managed for optimum yield, without any stresses deliberately imposed, and under generally favorable conditions (Supplementary Table S1). These trials are called stability trials and comprise a fairly consistent set of varieties over the years (strong carry-over from year to year), with newly released varieties added each year and hardly any varieties dropping out. The varieties included in the trials were developed by BRRI and representatives of all varieties that are grown in Bangladesh for both seasons. Thus, these trials form an ideal basis for assessing long-term genetic trends for yield achieved by BRRI breeding programs. This paper reports genetic trend in favorable environments of varieties developed both for favorable and less favorable environments.

**Fig. 1:**
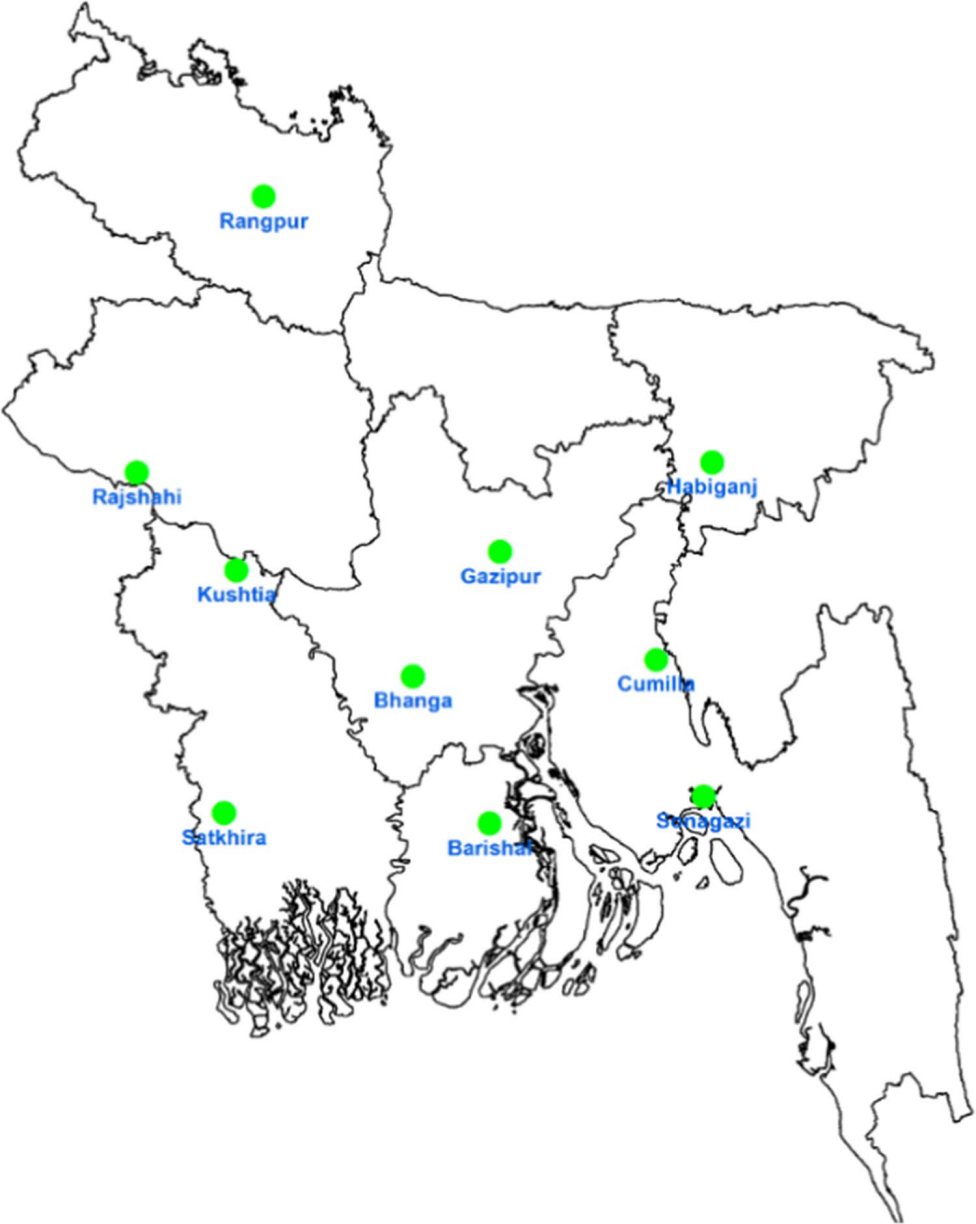
Multi-location rice variety trials from 2001 till 2020

The monsoon rice dataset contains the results of trials through 2020, while the winter rice dataset contains results through the growing season of 2020–2021. For each season, trials were conducted each year at nine locations for winter rice and eight locations for monsoon rice (Fig. 1).

### Rice varieties

**Fig. 2:**
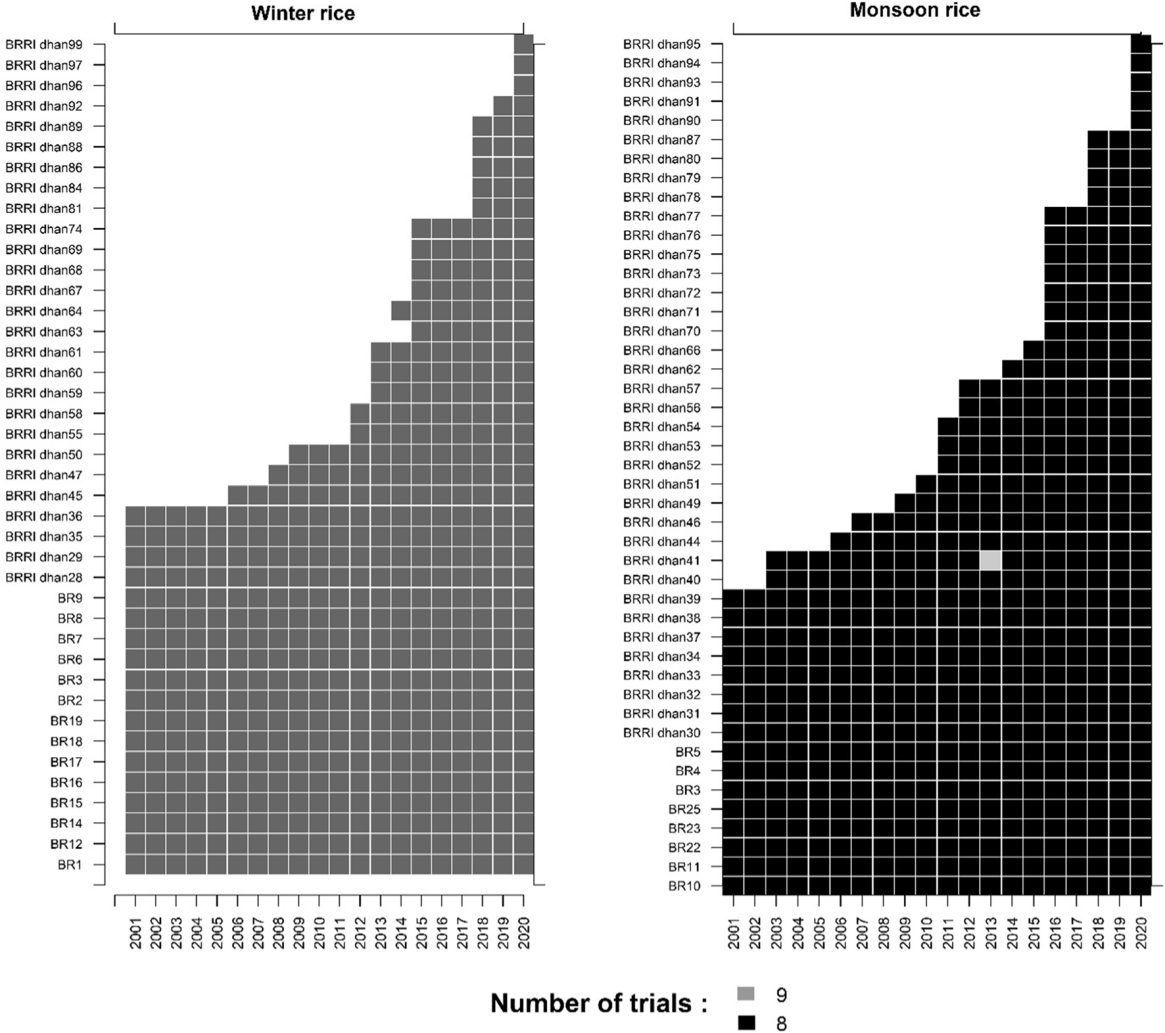
Classification of genotype-year combinations for winter rice series (left) and monsoon rice series (right). Cell shades of gray indicate number of trials in a year (color figure online)

From 1970 to 2020, BRRI released 41 varieties for the winter season and 45 varieties for the monsoon season. The first variety, BR1, was released in 1970 and the latest variety, BRRI dhan99, was released in 2020. A list of released varieties for both seasons is given in Table 1 (for details see Supplementary Table S1). As new varieties were added to the trials each year and old ones were retained, the number of tested varieties increased over the years. The genotype-year combinations for both seasons are shown in Fig. 2.

**Table 1:** List of rice varieties released between 1971 and 2020 in Bangladesh. Forty-one varieties released for winter season and 45 varieties released for monsoon season

Year of release	Variety	Year of release	Variety
	*Winter season*		*Monson season*
1970	BR1	1973	BR3
1971	BR2	1975	BR4
1973	BR3	1976	BR5
1977	BR6, BR7, BR8	1980	BR10, BR11
1978	BR9	1988	BR22, BR23
1983	BR12, BR14, BR15, BR16	1992	BR25
1985	BR17, BR18, BR19	1994	BRRI dhan30, BRRI dhan31, BRRI dhan32
1994	BRRI dhan28, BRRI dhan29	1997	BRRI dhan33, BRRI dhan34
1998	BRRI dhan35, BRRI dhan36	1998	BRRI dhan37, BRRI dhan38
2005	BRRI dhan45	1999	BRRI dhan39
2007	BRRI dhan47	2003	BRRI dhan40, BRRI dhan41
2008	BRRI dhan50	2005	BRRI dhan44
2011	BRRI dhan55	2007	BRRI dhan46
2012	BRRI dhan58	2008	BRRI dhan49
2013	BRRI dhan59, BRRI dhan60, BRRI dhan61	2010	BRRI dhan51, BRRI dhan52, BRRI dhan53, BRRI dhan54
2014	BRRI dhan63, BRRI dhan64, BRRI dhan67, BRRI dhan68, BRRI dhan69	2011	BRRI dhan56, BRRI dhan57
2015	BRRI dhan74	2013	BRRI dhan62
2017	BRRI dhan81, BRRI dhan84, BRRI dhan86	2014	BRRI dhan66
2018	BRRI dhan88, BRRI dhan89	2015	BRRI dhan70, BRRI dhan71, BRRI dhan72, BRRI dhan73
2019	BRRI dhan92	2016	BRRI dhan75, BRRI dhan76, BRRI dhan77, BRRI dhan78
2020	BRRI dhan96, BRRI dhan97, BRRI dhan99	2017	BRRI dhan79, BRRI dhan80
		2018	BRRI dhan87
		2019	BRRI dhan90, BRRI dhan91, BRRI dhan93, BRRI dhan94, BRRI dhan95

### Experiment and trial management

The experiments were set up as randomized complete block designs (RCBD) with three replicates in each location. For all varieties, breeder’s seed was used. In each location, 40-day-old seedlings for winter rice and 25-day-old seedlings for monsoon rice were transplanted. All transplanting was conducted manually, and any damaged seedlings were retransplanted by the same aged seedlings within 3–7 days of the first transplanting (see Table 2). The harvested area was 6 m^2^ out of 10 m^2^ per plot. The spacing between rows and between plants within row was 20 cm and 20 cm with 2 or 3 plants hill^−1^. There were 25 hills per m^2^, hence 6 m^2^ contained 150 hills. The fields were irrigated 4 days after transplanting and a water depth of approximately 5 to 10 cm was maintained until 7 days before physiological maturity. Seven days after transplanting, the missing or damaged hills were retransplanted with the same aged seedlings. Fungicides and pesticides were also applied to control major diseases and insects prevailing in the trials. Hand weeding was practiced as frequently as needed. The crop was harvested manually and border rows were not harvested. Grain yield (t ha^−1^) data were assessed discarding borders, amounting to a harvested area of about 2.72 m^2^ plot^−1^ and adjusted to a moisture content of 14%. The trial data are complete for all year-location combinations.

**Table 2:** Trial information for two rice seasons

Season	Seeding period	Transplanting period	Harvest period	Fertilizer application (kg/ha)				
				Urea	TSP^*^	MoP^*^	Gypsum	Zinc
Winter rice	15 Nov–30 Nov	25 Dec–10 Jan	25 April–14 May	160, 160, 140	52, 52, 48	88, 88, 80	60, 60, 60	6, 6, 6
Monsoon rice	15 June–30 June	15 Jul–30 Jul	15 Nov–30 Nov	104	32	56	36	

*TSP triple superphosphate, *MoP* muriate of potash

## Statistical analysis

### Analysis of genetic and non-genetic trends

In multi-environment trials, the analysis should ideally allow the variances to differ between year-location combinations. However, fitting such a model can become computationally demanding or even unfeasible due to memory issues. Therefore, a weighted two-stage analysis was applied here to allow for different error variances between year-location combinations. It is shown in Damesa et al. (2017) that the results of the two-stage analysis are usually very similar to those of a single-stage analysis.

In the first stage, the linear model (1) was fitted to the data of each year-location (environment) combination to estimate the genotype means and their associated standard errors:

1 $${y}_{il}=\mu +{b}_{l}+{g}_{i}+{e}_{il}$$

where ${y}_{il}$ is the observation of the *i*th genotype in the *l*th block in a given year-location combination, $\mu$ is a fixed intercept, ${b}_{l}$ is the fixed effect of the *l*th block, ${g}_{i}$ is the fixed effect of the *i*th genotype, and ${e}_{il}$ is the error associated with ${y}_{il}$ which is assumed to be independent and identically normally distributed.

In the second stage, following Piepho et al. (2014), linear mixed model (2) was fitted to the genotype means computed in the first stage:

2 $${y}_{ijk}=\mu +\beta {r}_{i}+{\gamma t}_{k}+{G}_{i}+{L}_{j}+{Y}_{k}+{\left(LY\right)}_{jk}+{\left(GL\right)}_{ij}+{\left(GY\right)}_{ik}+{\left(GLY\right)}_{ijk}+{e}_{ijk}$$

where ${y}_{ijk}$ is the mean yield of the *i*th genotype in the *j*th location and *k*th year, $\mu$ is a fixed intercept, $\beta$ is the fixed slope for genetic trend, ${r}_{i}$ is the year of release for the *i*th variety, $\gamma$ is the fixed slope for non-genetic trend, ${t}_{k}$ is the trial calendar year corresponding to the *k*th year, ${G}_{i}$ is the random main effect of the *i*th genotype, ${L}_{j}$ is the random main effect of the *j*th location, ${Y}_{k}$ is the random main effect of the *k*th year, ${\left(LY\right)}_{jk}$ is the *jk*th random location-year interaction effect, ${\left(GL\right)}_{ij}$ is the *ij*th random genotype-location interaction effect, ${\left(GY\right)}_{ik}$ is the *ik*th random genotype-year interaction effect, ${\left(GLY\right)}_{ijk}$ is the *ijk*th random genotype-location-year interaction effect, and ${e}_{ijk}$ is a random error and assumed independent and identically normally distributed. In model (2), the variance ${\sigma }_{{e}_{ijk}}^{2}$ of ${e}_{ijk}$ is assumed to be known and equal to the variance (squared standard error) of the corresponding genotype-location-year mean estimated from the analyses of variance of the individual trials computed in the first stage modeling complete blocks as fixed effects (Möhring and Piepho 2009, method 2). Variance components of the other random effects are denoted as ${\sigma }_{Y}^{2}$, ${\sigma }_{L}^{2}$, ${\sigma }_{G}^{2}$, ${\sigma }_{LY}^{2}$, ${\sigma }_{GY}^{2}$, ${\sigma }_{GL}^{2}$ and ${\sigma }_{GLY}^{2}$.

### Evaluation of trends

To estimate the genetic trend of yield, we computed the difference between the expected performance of the oldest variety in the first year of the trial series (2001 for both seasons) and the expected performance of the youngest variety in the last year of the trial series (2020 in winter season and 2020–2021 in monsoon season), ${y}_{1}$. Formally, the overall gain is estimated by

3 $$\mathrm{overall\; gain}=\frac{{y}_{1}-{y}_{0}}{{y}_{0}}=\frac{\mu +{\gamma t}_{max}+\beta {r}_{max}-\left(\mu +{\gamma t}_{min}+\beta {r}_{min}\right)}{\mu +{\gamma t}_{min}+\beta {r}_{min}}=\frac{\gamma \left({t}_{max}-{t}_{min}\right)+\beta \left({r}_{max}-{r}_{min}\right)}{\mu +{\gamma t}_{min}+\beta {r}_{min}}$$

where ${t}_{min}$ and ${t}_{max}$ are the first and the last calendar year of the trial series. The value of ${t}_{min}$ is 2001 and ${t}_{max}$ is 2020 for both seasons. Accordingly, ${r}_{min}$ and ${r}_{max}$ represent the year of release of the oldest and the youngest varieties, where ${r}_{min}$ is 1970 and 1973 and ${r}_{max}$ is 2020 and 2019 for winter and monsoon season, respectively. As the numerator of the total gain is additive, it can be separated into

4 $$\text{genetic gain}=\frac{\beta \left({r}_{max}-{r}_{min}\right)}{\mu +{\gamma t}_{min}+\beta {r}_{min}}$$

5 $$\text{non-genetic gain}=\frac{\gamma \left({t}_{max}-{t}_{min}\right)}{\mu +{\gamma t}_{min}+\beta {r}_{min}}$$

The gains per year due to genetic and non-genetic causes were estimated as

6 $$\text{Yearly }\text{genetic gain} =\frac{\beta }{\mu +{\gamma t}_{min}+\beta {r}_{min}}$$

7 $$\text{Yearly non-genetic gain}=\frac{\gamma }{\mu +{\gamma t}_{min}+\beta {r}_{min}}$$

where *μ* is the overall intercept, *β* and *γ* are the regression coefficients for genetic and non-genetic trend, respectively, and the start year (*t*_*min*_, *r*_*min*_) corresponds to the start of the period over which gain was assessed. To assess the gain for the last 50 years for both seasons, the value of *t*_*min*_ and *r*_*min*_ are 1970 for the winter season, and 1973 for the monsoon season, which are the release year of first variety for that season.

### Contribution of variance components to the average marginal variance

For each variance component of a model, its contribution to the average variance was evaluated by the ratio of a variance component to the mean total variance of the data. Due to the two-stage analysis, the second stage is ideally done treating the variances of the genotype means estimated in the first stage as known in the second stage. With these settings, the average marginal variance of the data is

8 $$AMV={\sigma }_{Y}^{2}+{\sigma }_{L}^{2}+{\sigma }_{G}^{2}+{\sigma }_{LY}^{2}+{\sigma }_{GY}^{2}+{\sigma }_{GL}^{2}+{\sigma }_{GLY}^{2}+\frac{1}{n}\sum {\sigma }_{{e}_{ijk}}^{2},$$

where $n$ is the number of genotype means estimated in the first stage and $\frac{1}{n}\sum {\sigma }_{{e}_{ijk}}^{2}$ is the mean variance of the genotype means. The contribution of a variance component to the average marginal variance was computed as

9 $${contribution}_{{\sigma }_{E}^{2}}=\frac{{\sigma }_{E}^{2}}{AMV}100\text{\%}$$

where ${\sigma }_{E}^{2}$ is one of the terms in $AMV$.

### Heritability

Heritability is often used by plant breeders as a measure of precision of a series of trials (Piepho and Möhring 2007). Heritability on a variety mean basis can be estimated according to Cullis et al. (2006) by

10 $${H}^{2}=1-\frac{mvd}{2{\sigma }_{g}^{2}}$$

where ${\sigma }_{g}^{2}$ is the genetic variance and $mvd$ is the mean variance of pairwise differences between estimated genotype effects (BLUPs). Both ${\sigma }_{g}^{2}$ and $mvd$ were obtained using (2).

## Results

**Fig. 3:**
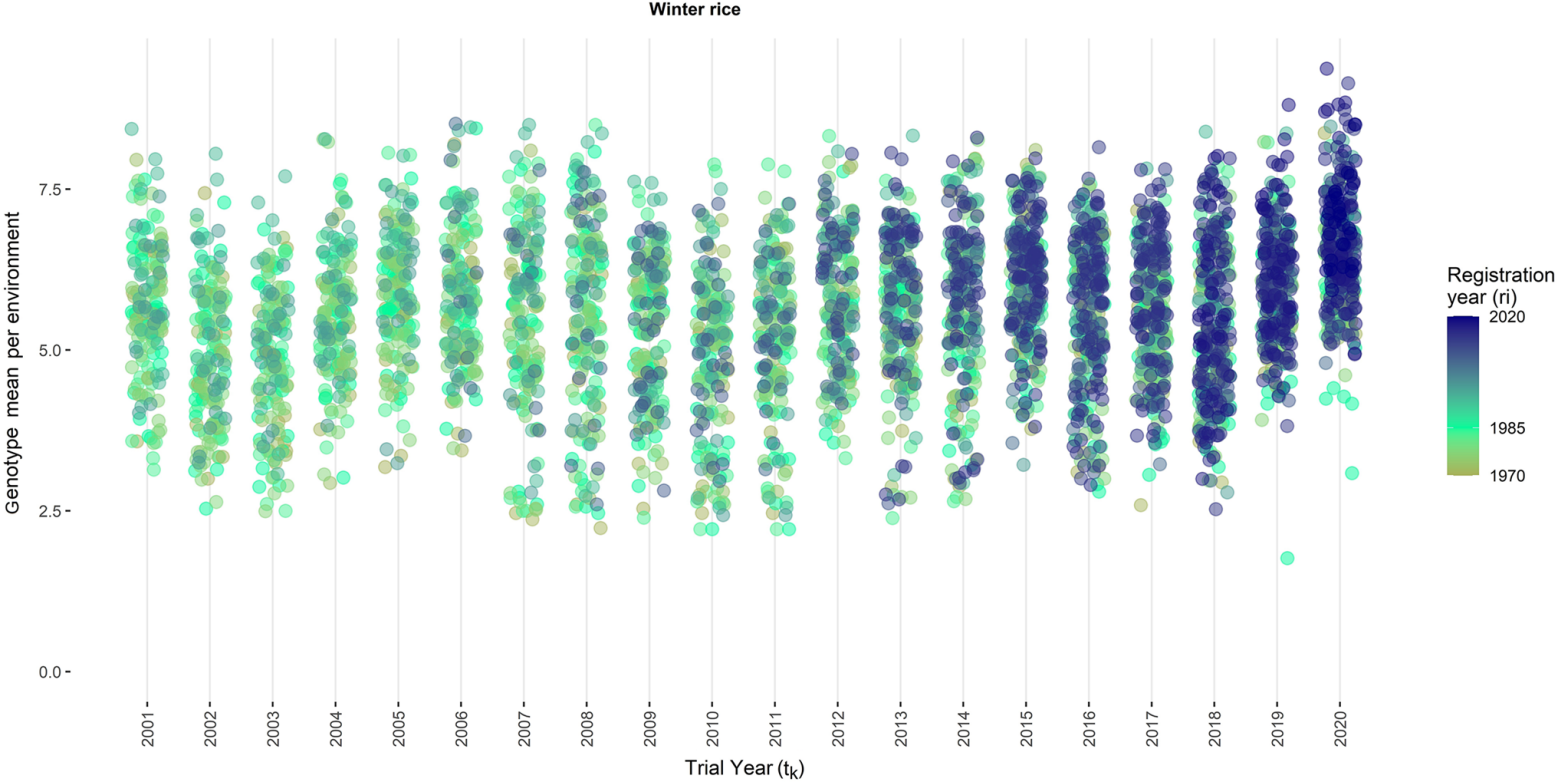
Genotype means per environment of winter rice seasons plotted against the trial year of the respective genotype. Colors indicate the year of release for the respective genotype (color figure online)

**Fig. 4:**
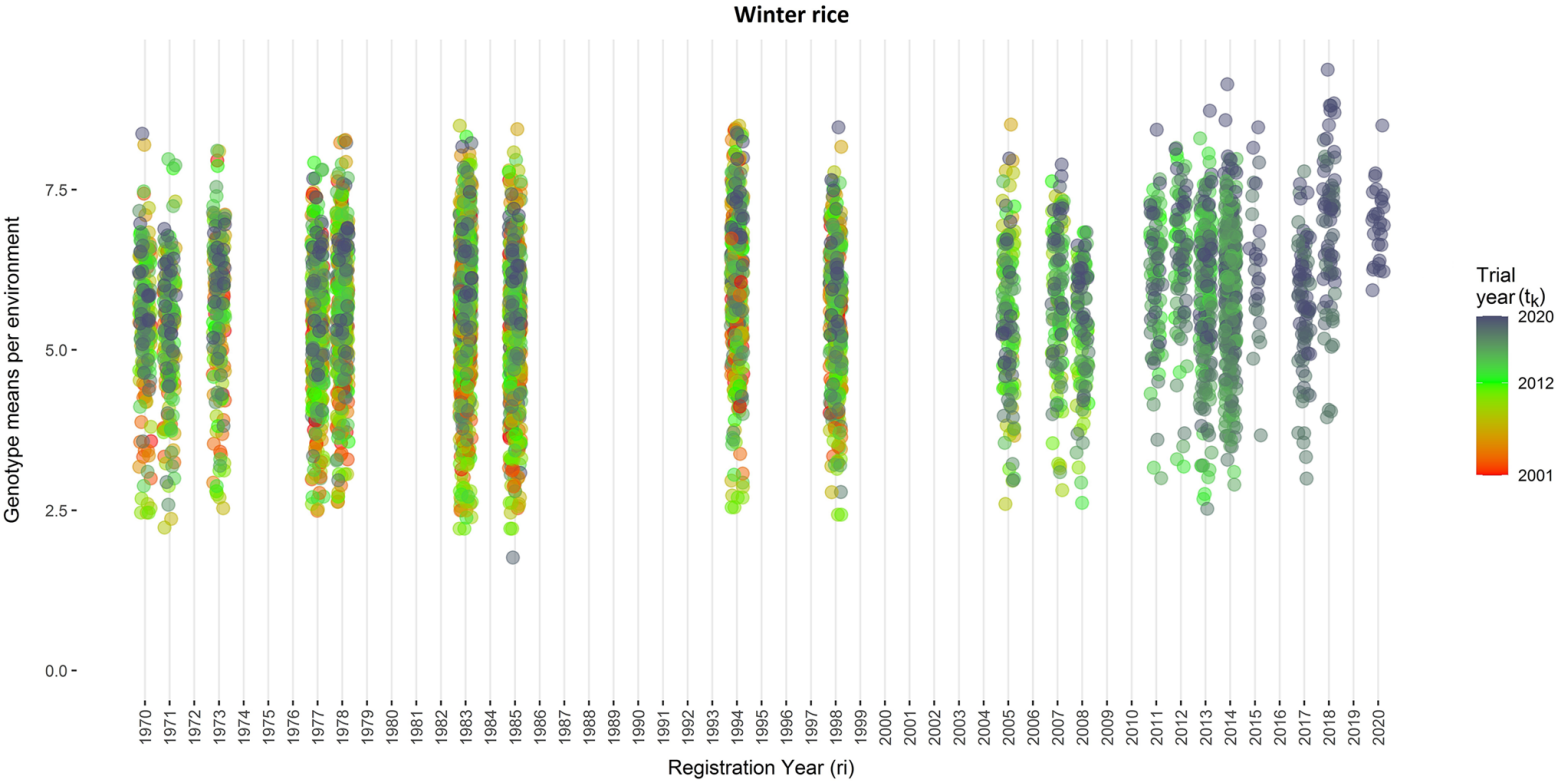
Genotype means per environment of winter rice seasons plotted against year of release for the respective genotype. Colors indicate the calendar year of the respective trial (color figure online)

**Fig. 5:**
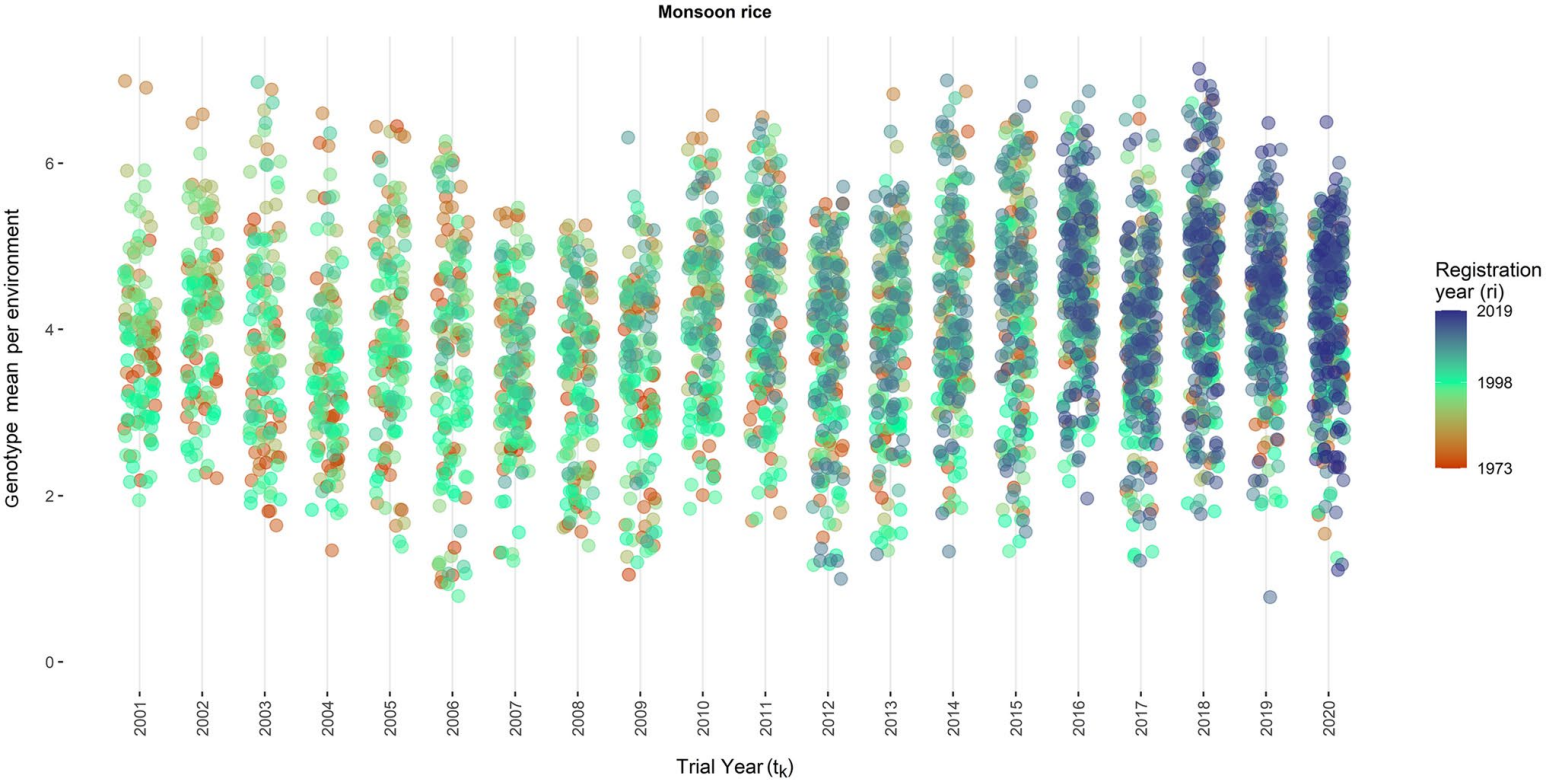
Genotype means per environment of monsoon rice seasons plotted against the trial year of the respective genotype. Colors indicate the year of release for the respective genotype (color figure online)

**Fig. 6:**
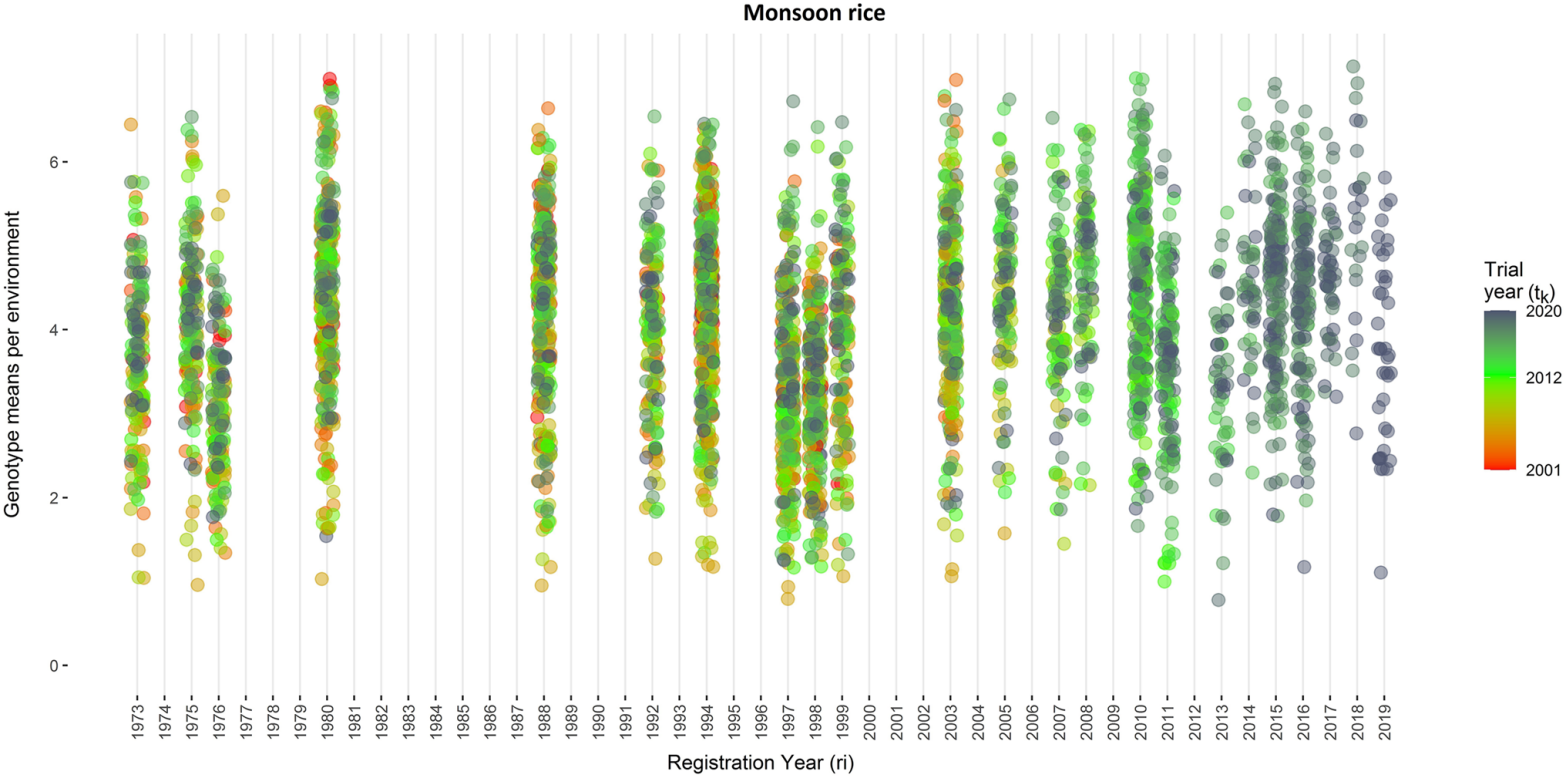
Genotype means per environment of monsoon rice seasons plotted against the year of release for the respective genotype. Colors indicate the calendar year of the respective trial (color figure online)

The estimated genotype-location-year means from Model (1) are plotted against the trial year and the release year of a variety in Figs. 3, 4, 5 and 6. The range of the genotype-location-year yields of winter rice varieties was between 1.76 t ha^−1^ and 9.37 t ha^−1^ (Fig. 3). The yield of monsoon rice varieties varied between 0.78 t ha^−1^ and 7.17 t ha^−1^ (Fig. 5). The newly registered varieties in winter season have higher yield as compared to older varieties. However, monsoon rice varieties have consistent yield over time.

The estimate of heritability for the winter rice and monsoon rice is 0.866 and 0.915, respectively. The high heritability estimates indicate the good reliability of the data.

### Genetic and non-genetic trends

The genetic trend was positive, with an increase in yield at the rate of 0.01 t/ha (confidence interval, CI: 0.006, 0.019) per year for winter rice (numbers in parenthesis indicate the lower and upper confidence limits at *α* = 0.05). The genetic trend for monsoon rice was also 0.01 t/ha (confidence interval, CI: −0.006, 0.017) per year (Table 3).

**Table 3:** Estimates and 95% confidence limits (CL) of intercepts ($\mu$), genetic trends ($\beta$) and non-genetic trend $(\gamma )$ of winter and monsoon rice varieties evaluated during the trial conducted from 2001 to 2020

Fixed effect	Winter rice			Monsoon rice		
	Estimate (S.E.)	Lower CL	Upper CL	Estimate (S.E.)	Lower CL	Upper CL
Intercept $(\mu )$	− 66.48 (27.08)	− 119.58	− 13.38	− 47.32 (25.01)	− 96.35	1.70
Genetic trend $(\beta )$	0.01 (0.003)	0.006	0.019	0.01 (0.006)	− 0.006	0.017
Non-genetic trend $(\gamma )$	0.02 (0.013)	− 0.002	0.049	0.02 (0.011)	− 0.002	0.042

The non-genetic trend was positive with an increase of 0.02 t/ha (confidence interval, CI: −0.002, 0.049) per year for winter rice, and 0.02 t/ha (confidence interval, CI: −0.002, 0.042) per year for monsoon rice.

The overall percentage change from 1970 till 2020 for winter rice was 40.96%, of which 13.91% was genetic gain and 27.05% non-genetic gain. Overall percentage change for monsoon rice was 38.39%, slightly less than for winter rice. The genetic gain was 8.36%, while non-genetic gain was 30.03% for monsoon rice (Table 4).

**Table 4:** Overall and yearly genetic and non-genetic gains (%)

	Winter rice		Monsoon rice	
	Total gain (%) (1970 to 2020)	Yearly gain (%)	Total gain (%) (1973 to 2020)	Yearly gain (%)
Overall gain	40.96	0.82	38.39	0.82
Genetic gain	13.91	0.28	8.36	0.18
Non-genetic gain	27.05	0.54	30.03	0.64

### Variance components

The variance components and the contribution to the total variance for location-year interaction effects for both seasons were relatively large compared to other variances (Table 5). The genotypic variance component observed for yield of both seasons was larger than that of the genotype-year and the genotype-location variance components suggesting substantial genetic variability and consistency of ranking across trials in the varieties used, indicative of limited genotype-environment interaction. However, the genotype-location-year variance in both seasons is larger than the other genotype-environment variances, and as large as or larger than the genotypic variances in both seasons.

**Table 5:** Estimates of variance components, standard error (S.E.) and the contribution of variance components to mean variance (%)

Variance component	Winter rice			Monsoon rice		
	Estimate	S.E.	Contribution (%)	Estimate	S.E.	Contribution (%)
*Genetic effects*						
${G}_{i}$	0.115	0.030	7.93	0.262	0.061	20.09
${\left(GY\right)}_{ik}$	0.018	0.004	1.26	0.017	0.004	1.28
${\left(GL\right)}_{ij}$	0.042	0.006	2.93	0.046	0.007	3.52
${\left(GLY\right)}_{ijk}$	0.254	0.008	17.48	0.270	0.008	20.64
*Non-genetic effects*						
${Y}_{k}$	0.050	0.039	3.44	0.017	0.028	1.26
${L}_{j}$	0.327	0.179	22.57	0.134	0.086	10.29
${\left(LY\right)}_{jk}$	0.552	0.065	38.08	0.491	0.062	37.58
${e}_{ijk}$	0.092		6.32	0.070		5.34

The variance components of ${e}_{ijk}$ represent the mean variance of the genotype means estimated in the first stage

## Discussion

In rice, there has been very limited research on genetic gain in Asia in general, and in Bangladesh, no studies have been done yet, even though rice is the main staple food. The aim of this study was to estimate the genetic gain for yield from the varieties released over the last 50 years for monsoon and winter rice ecosystems. The findings showed that over the fifty years from 1970 to 2020, the overall yield gain for winter rice was 40.96%, with an increase of 0.82% per year. The genetic gain for winter rice was 0.01 t ha^−1^ (0.28%) per year. The overall genetic improvement in yield over fifty years was 13.91%. The overall non-genetic percentage change in 50 years is 27.05%. Non-genetic gain for winter rice was 0.02 t ha^−1^ per year (0.54% per year), which is larger than genetic gain (Tables 3 and 4).

For monsoon rice, the overall yield gain over the 47 years from 1973 to 2020 was 38.39%, with an increase of 0.82% per year. The genetic gain was 0.01 t ha^−1^ per year, which is 0.18% change per year. The overall genetic percentage change in 47 years is 8.36%. Non-genetic gain for winter rice was 0.02 t ha^−1^ per year (0.64% per year). The overall non-genetic gain in 47 years is 30.03%, which is larger than the genetic gain (Tables 3 and 4).

Overall, genetic and non-genetic trend were quite similar for monsoon and winter rice, with non-genetic trend substantially exceeding genetic trend. Rates of genetic improvement for grain yield are low compared to some genetic gain estimates reported in rice for other programs (Peng et al. 2000; Tabien et al. 2008; McKenzie et al. 2014; Zhu et al. 2016; Breseghello et al. 2011), but not relative to gains reported by IRRI for favorable rainfed and irrigated environments. For India, Kumar et al. (2021) observed yearly genetic yield increases of 0.68% under irrigated conditions, 0.87% under moderate reproductive stage drought conditions, and 1.9% under acute reproductive stage drought conditions. Juma et al. (2021) estimated breeding values for rice grain yield using a mixed model approach considering the pedigree-based relationship matrix, which varied from 2.12 to 6.27 t ha^−1^. In the period 1964–2014, the average genetic gain of grain yield was 0.01 t ha^−1^ year^−1^ (0.23%). When only IRRI developed cultivars were considered in analysis, the rate rose to 0.02 t ha^−1^ year^−1^ (0.46%). Peng et al. (2000) assessed the trend in rice yield of IRRI cultivars released during 1966–1996, observing a gain of 1% per year. Muralidharan et al. (2002) examined the grain yield trends of rice cultivars developed and tested during 1976–1997 in METs conducted worldwide under various ecosystems in international rice advancement trials, concluding that there was no scientific proof for either a genetic gain or yield loss of genotypes released for any of the ecosystems. Muralidharan et al. (2019) estimated the genetic gain for yields in genotypes evaluated in 11 rice ecosystems in India during 1995–2013 and found a considerable gap between projected growth in human population and growth in national yield of rice. To ensure rice supply to meet the demands of a rising population, integrated genetic technology and policy interventions are required.

Overall, the rates of genetic gain achieved in monsoon and winter rice by the BRRI program from 1970 through 2020 have been quite low. These low rates of gain are consistent with those achieved by IRRI in favorable environments in the Philippines, but inadequate to keep pace with population growth, climate change, and loss of land due to urbanization. The rate of genetic gain can be improved by increasing selection differential with sufficient accuracy and decreasing cycle time (Cobb et al. 2019). In general, low rates of genetic gain in South and Southeast Asian rice breeding are likely mainly due to long breeding cycles caused by repeated use of older, popular varieties as parents, and by limited selection intensity for yield in multi-location trials. An analysis of the BRRI breeding program, conducted as part of a breeding modernization project initiated in 2015, indicated that both of these problems were affecting breeding progress. Key areas of weakness detected by BRRI in its review of its program included inadequate multi-location testing, inadequate selection intensity, and very long breeding cycles due to over-use of old parents. In the breeding program for the highly productive irrigated winter rice crop, the most popular varieties BRRI dhan28 and BRRI dhan29 were repeatedly used as parents, which resulted in limited improvement in additive breeding value. The key weaknesses of the BRRI breeding program that limit the rate of genetic gain are not unusual and affect many public sector breeding programs.

The current rate of genetic gain per year in rice yield of the BRRI released varieties of monsoon and winter season is only 0.01 t ha^−1^, which is not sufficient to meet the food requirements for the projected population of 215 million in 2050. The present rice production is about 38.7 million tons, whereas the population in Bangladesh is growing annually by 1.22%, arable land is decreasing annually by 0.4%, and climate vulnerability is also increasing. According to Kabir et al. (2015), genetic gain of at least 0.044 t ha^−1^ per year (approximately 1% annually) will be needed to meet Bangladesh’s requirements through 2050. Achieving this rate of genetic gain will be a challenging job for breeders, requiring a new strategy that involves accelerating the breeding cycle and shifting away from use of a few popular varieties and non-elite breeding pools as parents.

BRRI’s breeding programs are being reorganized and accelerated based on optimization strategies derived from application of the breeder’s equation to breeding pipeline design (Cobb et al. 2019; Atlin and Econopouly 2022). In recent years, BRRI’s project “Transforming Rice Breeding” reshaped the breeding programs to shorten the breeding cycle time, increase selection accuracy and intensity, and improve selection for breeding value. Breeding cycle time has been reduced by implementing single-seed descent and increasing the number of generations of advance annually from one to two or three, and by selecting parents after only one or two stages of replicated agronomic testing. Selection accuracy has been increased substantially by introducing multi-location testing at the first agronomic testing step (Stage 1), a practice which is not yet common in South Asian rice breeding programs. In most BRRI pipelines, the number of entries included in Stage 1 testing has increased seven to tenfold in the last five years. A trait development pipeline has now also been established parallel to the breeding pipeline to develop elite donors with increased frequency of alleles with large, well-validated effects on tolerance to biotic and abiotic stresses. Forward breeding became routine since early 2016 with the inception of the BRRI modernization project. Forward breeding with trait-specific SNP markers is also in routine use in BRRI breeding programs. Some of the breeding programs have already applied outsourced genome-wide marker systems to facilitate parent selection based on genomic estimated breeding values. When all these improvements are fully implemented, they are expected to result in a substantial improvement in the rate of genetic gain delivered by BRRI’s breeding programs.

The long-term trials summarized in this study are an important data resource for dissecting genetic and non-genetic trend in rice yields in South Asia. The similarity of non-genetic trend estimates for monsoon and winter yields may indicate that some common agronomic factors affected both seasons.

## Conclusion

This is the first time that genetic trend has been estimated for Bangladeshi rice breeding programs, and the estimates are among the few to have been published for South Asia. The results indicate that rice yield gains due to breeding have been very limited since the end of the Green Revolution in the favorable environments in which the experiment was conducted, amounting to 0.28% and 0.18% annually in irrigated winter and rainfed monsoon rice, respectively. These gains are lower than the non-genetic trend detected (0.54% and 0.64% annually for winter and monsoon rice, respectively) and are less than needed to maintain rice food security in the face of population growth, climate change, and land loss to urbanization. The low rates of genetic gain observed in this study appear to be broadly representative of those achieved in the favorable rice production environments in South Asia (Muralidharan et al. 2019) critical to the region’s food security, although there is some evidence that the rate of gain has been higher in drought-prone environments (Kumar et al. 2021).

Modest continuing gains in non-genetic trend of 0.54% and 0.64% annually in winter and monsoon, respectively, may be due to long-term improvements in crop management, but also may shed light on the impacts to date of climate change on rice productivity in favorable rice production environments in Bangladesh. Increasing temperatures are expected to increase climate risk to yields in monsoon production and decrease them in winter rice production (Sarker et al. 2017), however, we cannot dissect the effect of agronomic practices and impact of climate change.

The findings of this study confirm the need to increase the rate of genetic gain delivered by rice breeding programs in Bangladesh. Limiting factors have been carefully analyzed, and a program is designed to transform rice breeding at BRRI by reducing cycle time, increasing selection accuracy, and improving selection for breeding value. This program, technically supported by the International Rice Research Institute, is expected to increase the annual rate of genetic gain for yield to 1.5–2.0% in the near future.

We hope that the findings presented here will assist governments and policymakers in achieving higher rates of genetic improvement from rice breeding in South Asia. Efforts are needed both to improve breeding pipelines and to accelerate dissemination of newly developed improved varieties and withdrawal of old varieties. Now that a high level of fertilizer and crop protection input use has been attained in Bangladeshi rice production, higher rates of genetic improvement are likely to be the principal pathway to sustaining rice food security.

## Supplementary Information

Below is the link to the electronic supplementary material.

Supplementary file1 (DOCX 21 kb)

## Declarations

### Data availability

The datasets generated during and/or analyzed during the current study are available on reasonable request.

## References

[CR1] Ahrends HE, Eugster W, Gaiser T, Rueda-Ayala V, Hüging H, Ewert F, Siebert S (2018). Genetic yield gains of winter wheat in Germany over more than 100 years (1895–2007) under contrasting fertilizer applications. Environ Res Lett.

[CR2] Atlin GN, Econopouly BF (2022). Simple deterministic modeling can guide the design of breeding pipelines for self-pollinated crops. Crop Sci.

[CR3] BBS (Bangladesh Bureau of Statistics). (1972) Yearbook of Agricultural Statistics of Bangladesh 1972. Ministry of Planning, Government of the People’s Republic of Bangladesh.

[CR4] BBS (Bangladesh Bureau of Statistics). (2019) Yearbook of Agricultural Statistics of Bangladesh 2019. Ministry of Planning, Government of the People’s Republic of Bangladesh

[CR5] Bell AR, Bryan E, Ringler C, Ahmed A (2015). (2015) Rice productivity in Bangladesh: What are the benefits of irrigation?. Land Use Policy.

[CR6] Breseghello F (2011). Results of 25 years of upland rice breeding in Brazil. Crop Sci.

[CR7] Brolley, M. (2015) Rice security is food security for much of the world. Rice Today. International Rice Research Institute (IRRI), DAPO Box 7777, Metro Manila, Philippines. pp. 30–32

[CR8] Cobb JN, Juma RU, Biswas PS (2019). Enhancing the rate of genetic gain in public-sector plant breeding programs: lessons from the breeder’s equation. Theor Appl Genet.

[CR9] Cullis BR, Smith AB, Coombes NE (2006). On the design of early generation variety trials with correlated data. J Agr Biol Environ Stat.

[CR10] DAE (2020) Weekly crop production report. Department of agricultural extension, Ministry of agriculture, Government people's republic of Bangladesh, Dhaka, Bangladesh

[CR11] Damesa MT, Möhring J, Worku M, Piepho HP (2017). One step at a time: Stage wise analysis of a series of experiments. Agrono J.

[CR12] Duvick DN (2005). Genetic progress in yield of United States maize (Zea mays L.). Maydica.

[CR13] Duvick DN, Smith JSC, Cooper M (2004). Long-term selection in a commercial hybrid maize breeding program. Plant Breed Rev.

[CR14] FPMU (2020) Bangladesh Food Situation Report, April-June 2020. Food planning and monitoring unit, Ministry of food, Government People’s Republic of Bangladesh, Dhaka, Bangladesh. Vol. 121

[CR15] HIES (Household Income and Expenditure Survey), (2016) Bangladesh Bureau of Statistics. Government of Bangladesh, Dhaka

[CR16] Juma RU, Bartholomé J, Thathapalli Prakash P, Hussain W, Platten JD, Lopena V, Cobb JN (2021). Identification of an elite core panel as a key breeding resource to accelerate the rate of genetic improvement for irrigated rice. Rice.

[CR17] Kabir MS, Salam MU, Chowdhury A, Rahman NMF, Iftekharuddaula KM, Rahman MS, Rashid MH, Dipti SS, Islam A, Latif MA, Islam AKMS, Hossain MM, Nessa B, Ansari TH, Ali MA, Biswas JK (2015). Rice vision for Bangladesh: 2050 and beyond. Bangladesh Rice J.

[CR18] Kumar A, Raman A, Yadav S, Verulkar SB, Mandal NP, Singh ON, Swain P, Ram T, Badri J, Dwivedi JL, Das SP, Singh SK, Singh SP, Kumar S, Jain A, Chandrababu R, Robin S, Shashidhar HE, Hittalmani S, Piepho HP (2021). Genetic gain for rice yield in rainfed environments in India. Field Crops Res.

[CR19] Laidig F, Piepho HP, Drobek T, Meyer U (2014). Genetic and non-genetic long-term trends of 12 different crops in German official variety performance trials and on-farm yield trends. Theor Appl Genet.

[CR20] Laidig F, Piepho HP, Rentel D, Drobek T, Meyer U, Huesken A (2017). Breeding progress, variation, and correlation of grain and quality traits in winter rye hybrid and population varieties and national on-farm progress in Germany over 26 years. Theor Appl Genet.

[CR21] Mackay I, Horwell A, Garner J, White J, McKee J, Philpott H (2011). Reanalyses of the historical series of UK variety trials to quantify the contributions of genetic and environmental factors to trends and variability in yield over time. Theor Appl Genet.

[CR22] Masuka B, Atlin GN, Olsen M, Magorokosho C, Labuschagne M, Crossa J, Bänziger M, Pixley KV, Vivek BS, von Biljon A, Macrobert J (2017). Gains in maize genetic improvement in Eastern and Southern Africa: I CIMMYT hybrid breeding pipeline. Crop Sci.

[CR23] McKenzie K, Sha X, Moldenhauer K, Linscombe S, Lyman N, Nalley L, Smith S, Diers B, Specht J, Carver B (2014). Rice. Yield gains in major U.S. field crops.

[CR24] Mottaleb KA, Mishra AK (2016). Rice consumption and grain-type preference by household: a Bangladesh case. J Agric Appl Econ.

[CR25] Möhring J, Piepho HP (2009). Comparison of weighting in two-stage analysis of plant breeding trials. Crop Sci.

[CR26] Muralidharan K, Prasad CSV, Rao CS (2002). Yield performance of rice genotypes in international multi-environment trials during 1976–97. Curr Sci.

[CR27] Muralidharan K, Prasad CSV, Rao CS, Siddiq EA (2019). Genetic gain for yield in rice breeding and rice production in India to meet with the demand from increased human population. Curr Sci.

[CR28] Peng S, Laza R, Visperas R, Sanico A, Cassman K, Khush G (2000). Grain yield of rice cultivars and lines developed in the philippines since 1966. Crop Sci.

[CR29] Piepho HP, Laidig F, Drobek T, Meyer U (2014). Dissecting genetic and non-genetic sources of long-term yield trend in German official variety trials. Theor Appl Genet.

[CR30] Piepho HP, Möhring J (2007). Computing heritability and selection response from unbalanced plant breeding trials. Genetics.

[CR31] Rife TW, Graybosch RA, Poland JA (2019). A field-based analysis of genetic improvement for grain yield in winter wheat cultivars developed in the us central plains from 1992 to 2014. Crop Sci.

[CR32] Sarker Md AR, Alam K, Gow J (2017). Performance of rain-fed Aman rice yield in Bangladesh in the presence of climate change. Renew Agric Food Syst.

[CR33] Tabien R, Samonte S, McClung A (2008). Forty-eight years of rice improvement in Texas since the release of cultivar Bluebonnet in 1944. Crop Sci.

[CR34] World Bank (2016) https://www.worldbank.org/en/results/2016/10/07/bangladesh-growing-economy-through-advances-in-agriculture

[CR36] Xu Y, Li P, Zou C, Lu Y, Xie C, Zhang X, Prasanna BM, Olsen MS (2017). Enhancing genetic gain in the era of molecular breeding. J Exp Bot.

[CR35] Zhu G (2016). Genetic improvements in rice yield and concomitant increases in radiation- and nitrogen-use efficiency in middle reaches of Yangtze river. Sci Rep.

