# Supplementary material for: 50 years of rice breeding in Bangladesh: genetic yield trends
Source: Theor Appl Genet. 2023 Jan 21;136(1):18. doi: 10.1007/s00122-023-04260-x (PMC9867671; doi:10.1007/s00122-023-04260-x)
Supplement: Supplementary file 1 — Supplementary file1 (DOCX 21 kb) [file 122_2023_4260_MOESM1_ESM.docx]

# Supplementary

**Table S1a** Variety list in chronological order as per year of release for winter rice season

| Variety | Parentage | Target environment | Category | Year of release | Salient feature |
| --- | --- | --- | --- | --- | --- |
| BR1 | IR262-24-3/TKM6 | Favorable | Medium | 1970 | Bold and short grain type |
| BR2 | CP-SLO/Sigadis | Favorable | Long | 1971 | Medium slender and white grain type |
| BR3 | IR506-1-133/Latisail | Favorable | Long | 1973 | Medium bold grain and white belly |
| BR6 | IR833-6-2-1-1/IR1561-149-1/IR1737 | Favorable | Short | 1977 | Long, slender and white grain |
| BR7 | IR1416-131-5/IR22/C4-63 | Favorable | Medium | 1977 | Long, Slender grain |
| BR8 | IR272-4-1-2-J1/IR305-3-17-1-3 | Favorable | Long | 1977 | Medium slender and white belly, suitable for hailstorm |
| BR9 | IR272-4-1-2-J1/IR8 | Favorable | Medium | 1978 | Long, medium bold and white, suitable for hailstorm |
| BR12 | BR1/IR425-1-1-3-8-3 | Favorable | Long | 1983 | medium bold and white grain |
| BR14 | IR5 (D)/BR3 | Favorable | Long | 1983 | medium bold and white grain |
| BR15 | IR1561-228-1-2/IR1737/CR94-13 | Favorable | Long | 1983 | medium slender and white grain |
| BR16 | IR1416-131-5/IR1364-37-3-1/IR1544A-E666 | Favorable | Long | 1983 | Long slender and white |
| BR17 | Zerak/IR8 | Haor | Medium | 1985 | medium bold and suitable for Haor |
| BR18 | Pelita1-1 and IR1108-2 | Haor | Long | 1985 | medium bold, white and suitable for Haor |
| BR19 | IR2180-2/IR2178-1 | Haor | Long | 1985 | medium bold, white and suitable for Haor |
| BRRI dhan28 | BR6 (IR28)/Purbachi | Favorable | Short | 1994 | Medium slender and white grain |
| BRRI dhan29 | BG90-2/BR51-46-5 | Favorable | Long | 1994 | Medium slender and white grain |
| BRRI dhan35 | BR4/BR26-7-4-1/ARC14529 | Favorable | Medium | 1998 | Short and bold grain type, Brown plant hopper resistant |
| BRRI dhan36 | IR64/IR35293-125-3-2-3 | Cold | Short | 1998 | Long slender grain and cold tolerant |
| BRRI dhan45 | BR2/TETEP | Favorable | Short | 2005 | Medium bold and white |
| BRRI dhan47 | IR515111-B-B-34-B/TCCP266-2-49-B-B-3 | Salinity | Medium | 2007 | Medium bold and salt tolerant |
| BRRI dhan50 | BR30/IR67684B | Favorable | Medium | 2008 | Premium quality |
| BRRI dhan55 | IR64/*O. rufipogon* | Salinity | Short | 2011 | Long slender and moderately cold, drought, salinity tolerant |
| BRRI dhan58 | Somaclone of BRRI dhan29 | Favorable | Medium | 2012 | Slender and BRRI dhan29 grain type |
| BRRI dhan59 | -- | Favorable | Medium | 2013 | Medium bold and white, lodging resistant |
| BRRI dhan60 | BR7166-4-5-3/BR26 | Favorable | Medium | 2013 | Long slender and white grain |
| BRRI dhan61 | IR64419-3B-4-3/BRRI dhan29 | Salinity | Medium | 2013 | Medium slender, white and salinity tolerant |
| BRRI dhan63 | Amol-3/BRRI dhan28 | Favorable | Medium | 2014 | Long slender like slender balam |
| BRRI dhan64 | IR75382-32-2-3-3/BR7166-4-5-3-2-5-5B1-92 | Favorable | Medium | 2014 | Medium bold and white, High zinc enriched |
| BRRI dhan67 | IR61247-3B-8-2-1/BRRI dhan36 | Salinity | Short | 2014 | Medium slender grain, white, high amylose |
| BRRI dhan68 | BRRI dhan29*2/IR68144 | Favorable | Medium | 2014 | Medium bold and white grain |
| BRRI dhan69 | WuShanYouZhan/PI312777 | Favorable | Medium | 2014 | Medium bold and white grain |
| BRRI dhan74 | BRRI dhan29*2/IR68144 | Favorable | Medium | 2015 | Medium slender, white and zinc enriched (24.2 mg kg^-1^) |
| BRRI dhan81 | Amol-3/BRRI dhan28 | Favorable | Short | 2017 | Long slender, Zira type and high protein |
| BRRI dhan84 | BRRI dhan29/IR68144// BRRI dhan28///BR11 | Favorable | Short | 2017 | Like grain type of BRRI dhan28, high zinc (27.6 mg kg^-1^) and red coated |
| BRRI dhan86 | Niamat/BR802-78-2-1-1 | Favorable | Short | 2017 | Long slender |
| BRRI dhan88 | Somaclone of BRRI dhan29 | Favorable | Short | 2018 | Long slender and white color |
| BRRI dhan89 | BRRI dhan29*3/*Oryza rufipogon (IRGC 103404)* | Favorable | Long | 2018 | Medium bold and white color |
| BRRI dhan92 | Rice/Wheat(R1)/BR319-1-HR2//DH (Mingolo/Suweon290)/Panbira | Favorable (water saving) | Long | 2019 | Long slender |
| BRRI dhan96 | BRRI dhan28*3/*Oryza rufipogon (IRGC 103404)* | Favorable | Short | 2020 | Short bold, high amylose |
| BRRI dhan97 | IRRI113/BRRI dhan40 | Salinity | Medium | 2020 | Medium bold, white color |
| BRRI dhan99 | Huang-Hua-Zhan/OM1723 | Salinity | Medium | 2020 | Long slender, white color |

**Table S1b:** Variety list in chronological order as year^-1^ of release for monsoon rice season

| Variety | Parentage | Target environment | Category | Year of release | Salient feature |
| --- | --- | --- | --- | --- | --- |
| BR3 | IR506-1-133/Latisail | Favorable | Long | 1973 | Medium bold and white belly |
| BR4 | IR20/IR5-114-3-1 | Favorable | Long | 1975 | Medium bold |
| BR5 | Badshahbhog | Favorable | Long | 1976 | Small and round grain, aroma |
| BR10 | IR20/IR5-114-3-1 | Favorable | Long | 1980 | Medium slender grain |
| BR11 | IR20/IR5-47-2 | Favorable | Long | 1980 | Medium bold grain |
| BR22 | Nizersail/BR51-46-5 | Favorable | Long | 1988 | Short, bold and white, strongly photosensitive |
| BR23 | DA29/BR4 | Favorable | Long | 1988 | Long slender, white and late variety |
| BR25 | Pajam/IR26 | Favorable | Medium | 1992 | Short, bold and white grain |
| BRRI dhan30 | IR2058-78-1-3-2-3/BR4 | Favorable | Long | 1994 | Medium slender and white |
| BRRI dhan31 | BR11/ARC10550 | Favorable | Long | 1994 | Medium bold and white |
| BRRI dhan32 | BR4/BR2662 | Favorable | Medium | 1994 | Medium bold and white grain |
| BRRI dhan33 | BG388/BR2662 | Favorable | Short | 1997 | Short, Bold and white belly, early variety |
| BRRI dhan34 | Khaskani | Favorable | Medium | 1997 | Short, bold and aromatic |
| BRRI dhan37 | Basmati (D)/BR5 | Favorable | Long | 1998 | Medium slender and aroma |
| BRRI dhan38 | Basmati (D)/BR5 | Favorable | Long | 1998 | Long slender and aromatic |
| BRRI dhan39 | BR1185-2B-56-2-1-1/BR1674-28-3-1-1//BR2558-7-3-2-2 | Favorable | Medium | 1999 | Long sender grain |
| BRRI dhan40 | IR4595-4-1-15/BR10 | Salinity | Long | 2003 | Medium bold and salinity tolerant |
| BRRI dhan41 | BR23/BR1185-2B-16-1 | Salinity | Long | 2003 | Long bold and salinity tolerant |
| BRRI dhan44 | BR10/BRRI dhan31 | Submergence | Long | 2005 | Bold grain, tidal submergence tolerant |
| BRRI dhan46 | BR11/Swarnalata//ARC14766A | Favorable | Medium | 2007 | Medium bold, late variety |
| BRRI dhan49 | BR4962-12-4-1/IR33380-7-2-1-3 | Favorable | Medium | 2008 | Medium slender and Naizersail type |
| BRRI dhan51 | Swarna/IR49830-7-1-2-3 | Submergence | Long | 2010 | Medium bold and submergence tolerant |
| BRRI dhan52 | BR11/IR40931-33-1-3-2 | Submergence | Long | 2010 | Medium bold and submergence tolerant |
| BRRI dhan53 | BR10/BR23//BR847-76-1-1 | Salinity | Medium | 2010 | Medium bold, salinity tolerant |
| BRRI dhan54 | BR1185-2B-16-1/BR548-128-1-3 | Salinity | Medium | 2010 | Medium bold and salinity tolerant |
| BRRI dhan56 | Wayrarem/2*IR5519-4 | Favorable | Short | 2011 | Long bold, white and drought tolerant |
| BRRI dhan57 | BR11/5*CR146-7027-224 | Drought | Short | 2011 | Zirasail and Miniket type grain and drought tolerant |
| BRRI dhan62 | Zirakatari/BRRI dhan39 | Favorable | Short | 2013 | Long slender, white, medium zinc enriched (20 mg/kg), early variety |
| BRRI dhan66 | IR78875-176-B-2/IR78875-207-B-3 | Favorable | Short | 2014 | Medium long and bold and white grain |
| BRRI dhan70 | IR67423-208-6-2-3-3/IR65610-105-2-5-2-2-2 | Favorable | Medium | 2015 | Long slender with aroma |
| BRRI dhan71 | IR55423-01 (NSICRc9)/IRRI148 | Favorable | Short | 2015 | Medium long and drought tolerant |
| BRRI dhan72 | BR7166-4-5-3/BRRI dhan39 | Favorable | Medium | 2015 | Long, bold and white grain with zinc enriched (22.8 mg/kg) |
| BRRI dhan73 | BRRI dhan40/NSICRc106 (IR61920-3-B-22-2-1) | Favorable | Medium | 2015 | Medium slender and saline tolerant |
| BRRI dhan75 | Yuefengzhan/E-Zhong5 | Favorable | Short | 2016 | Long slender with some aroma after cooking |
| BRRI dhan76 | IR75862-208-8-B-B-HR1/BR6110-10-1-2 | Non-saline tidal flood | Long | 2016 | Medium bold and translucent |
| BRRI dhan77 | IR75862-208-8-B-B-HR1/BR6110-10-1-2 | Non-saline tidal flood | Long | 2016 | Medium bold and translucent |
| BRRI dhan78 | IR84645/IR84649 | Salinity | Medium | 2016 | Medium slender |
| BRRI dhan79 | BRRI dhan49*6/BRRI dhan52 | Submergence | Medium | 2017 | Medium slender, long and white color |
| BRRI dhan80 | IR65610-105-2-5-2-2/IR67423-208-6-2-3-3 | Favorable | Medium | 2017 | Narrow and long jasmine type with non-sticky |
| BRRI dhan87 | BRRI dhan29*3/*Oryza rufipogon (IRGC 103404)* | Favorable | Medium | 2018 | Long slender and white color |
| BRRI dhan90 | BR7166-5B-1-RAN-1/BRRI dhan34//BR7166-5B-1-RAN-1 | Favorable | Medium | 2019 | Small grain, very short bold, white color |
| BRRI dhan91 | Tilak kachari/BRRI dhan41 | Medium Deep water | Long | 2019 | Medium bold, light brown color |
| BRRI dhan93 | Pure line of Swarna-5 | Favorable | Medium | 2019 | Medium bold, white color |
| BRRI dhan94 | Pure line of Ranjit Swarna | Favorable | Medium | 2019 | Medium bold, white color |
| BRRI dhan95 | Lal Swarna//Barisail/PSBRc2 | Favorable | Medium | 2019 | Medium bold, white color |
